# Causal association between peripheral immune cells and IgA nephropathy: a Mendelian randomization study

**DOI:** 10.3389/fimmu.2024.1371662

**Published:** 2024-08-16

**Authors:** Li-Mei Liang, Liang Xiong, Xin-Liang He, Lin-Jie Song, Xiaorong Wang, Yu-Zhi Lu, Hong Ye, Wan-Li Ma, Fan Yu

**Affiliations:** ^1^ Department of Respiratory and Critical Care Medicine, Union Hospital, Tongji Medical College, Huazhong University of Science and Technology, Wuhan, China; ^2^ Key Laboratory of Respiratory Diseases, National Health Commission of China, Wuhan, China; ^3^ Department of Pulmonary and Critical Care Medicine, The Central Hospital of Wuhan, Tongji Medical College, Huazhong University of Science and Technology, Wuhan, China; ^4^ Department of Pathophysiology, School of Basic Medicine, Tongji Medical College, Huazhong University of Science and Technology, Wuhan, China

**Keywords:** causal relationship, immunoglobin A nephropathy, lymphocyte subsets, Mendelian randomization, peripheral immune cells

## Abstract

**Background:**

The relationship between peripheral immune cells and immunoglobulin A nephropathy (IgAN) is widely known; however, causal evidence of this link is lacking. Here, we aimed to determine the causal effect of peripheral immune cells, specifically total white blood cells, lymphocytes, monocytes, basophils, eosinophils, and neutrophils, as well as lymphocyte subset traits, on the IgAN risk using a Mendelian randomization (MR) analysis.

**Methods:**

The inverse-variance weighted (IVW) method was used for the primary analysis. We applied three complementary methods, including the weighted median, MR-Egger regression, and MR-PRESSO, to detect and correct for the effect of horizontal pleiotropy. Additionally, we performed a multivariable MR (MVMR) analysis, adjusting for the effects of C-reactive protein (CRP) levels. The roles of specific lymphocyte subtypes and their significance have garnered interest. Bidirectional two-sample MR analysis was performed to test the potential causal relationships between immune traits, including median fluorescence intensities (MFIs) and the relative cell count (AC), and IgAN.

**Results:**

The IVW-MR analysis suggested a potential causal relationship between lymphocyte counts and IgAN in Europe (OR per 1-SD increase: 1.43, 95% CI: 1.08–1.88, *P* = 0.0123). The risk effect of lymphocytes remained even after adjusting for CRP levels using the MVMR method (OR per 1-SD increase: 1.44, 95% CI: 1.05–1.96, *P* = 0.0210). The other sensitivity analyses showed a consistent trend. The largest GWAS published to date was used for peripheral blood immunophenotyping to explore the potential causal relationship between peripheral immune cell subsets and IgAN. Six AC–IgAN and 14 MFI–IgAN pairs that reached statistical significance (*P* < 0.05) were detected. Notably, CD3, expressed in eight subsets of T cells, consistently showed a positive correlation with IgAN. The bidirectional MR analysis did not reveal any evidence of reverse causality. According to the sensitivity analysis, horizontal pleiotropy was unlikely to distort the causal estimates.

**Conclusions:**

Genetically determined high lymphocyte counts were associated with IgAN, supporting that high lymphocyte counts is causal risk factor for IgAN.

## 1 Introduction

Immunoglobulin A nephropathy (IgAN) is a common immune-mediated kidney disease characterized by IgA deposition in the glomeruli (1). Globally, it is a primary glomerular disease and a leading cause of chronic kidney disease and renal failure. Despite this, there is currently no established disease-specific treatment available, and within 10–15 years of diagnosis, 40–50% of patients with IgAN develop kidney failure (1–3). IgAN is thus a disease that imposes a significant burden on patients and healthcare services worldwide.

The risk of developing IgAN and the likelihood of disease progression are influenced by both genetic and environmental factors (1). IgAN has the highest recorded prevalence in the Asia-Pacific region and Europe, accounting for 50% and 20% of all glomerular diseases, respectively. By contrast, IgAN is rare in South America and Africa. Geographical differences in the prevalence of IgAN could be attributed to varying frequencies of risk alleles. East Asian populations have the highest number of risk alleles, whereas African and African American populations have the lowest. Previous IgAN studies have identified independent disease-associated loci involving mucosal innate immunity, maintenance of the intestinal barrier, and the complement pathway using a genome-wide association study (GWAS) approach (4–7). The pathophysiology of IgAN remains unclear, but it is widely accepted that IgAN development and progression involve four main stages, known as the multi-hit hypothesis (8). The first hit involves the stimulation of mucosal innate immune cells, resulting in the production of large amounts of galactose-deficient IgA1 (Gd-IgA1) that enters the circulation (9). The second hit involves the production of IgG, IgA, and IgM antibodies against Gd-IgA1 in the bone marrow (10, 11). In the third hit, anti-Gd-IgA1 auto-antibodies form immune complexes with Gd-IgA1 and its aggregates, and these travel to the kidneys through blood vessels (12, 13). In the fourth hit, the accumulation of immune complexes in the glomerular mesangium triggers mesangial cell proliferation, complement activation, and immune cell recruitment, resulting in glomerular injury (14, 15). Recent studies have provided evidence supporting the view that immune dysregulation plays a prominent role in IgAN predisposition.

Routine blood tests are used to measure various subtypes of peripheral immune cells, including total white blood cells (WBCs), lymphocytes, monocytes, neutrophils, basophils, and eosinophils (16). Alterations in the circulating immune cell counts have been found to play a significant role in the pathogenesis, progression, severity, and prognosis of IgAN (17–19). For example, patients with IgAN have increased CD38(+) B cell and plasma cell numbers. These cells are thought to be responsible for the elevated production of Gd-IgA1 and anti-Gd-IgA1 antibodies (20). Additionally, patients with IgAN have higher levels of Tim-3-expressing circulating monocytes and macrophages than healthy controls, which could be involved in the development of IgAN (17). In patients with IgAN, a correlation between the neutrophil-to-lymphocyte ratio and poor renal outcomes, including a lower eGFR, increased proteinuria, and a lower rate of event-free renal survival, has also been established (18). However, causal evidence of a relationship between peripheral immune cells and IgAN is lacking.

Mendelian randomization (MR) analysis is an epidemiological approach for causal inference, in which genetic variants strongly associated with exposure factors are used as instrumental variables (IVs) (21, 22). MR functions similarly to a randomized controlled trial because genotypes are randomly allocated during meiosis, which can limit the influence of confounding factors and reverse causality (23). Hematopoiesis is a highly regulated process, with genetic variation leading to alterations in blood cell counts (24). Given that peripheral blood cell measurements are informative as intermediate phenotypic measures for disease assessment, we postulated that an investigation into the causal associations between circulating immune cell counts and IgAN may provide insights into the underlying etiologies of IgAN. In this study, a two-sample MR analysis was first performed to investigate the potential causal role of peripheral blood cell measurements in the risk of IgAN.

## 2 Materials and methods

### 2.1 Study design

**Figure 1:**
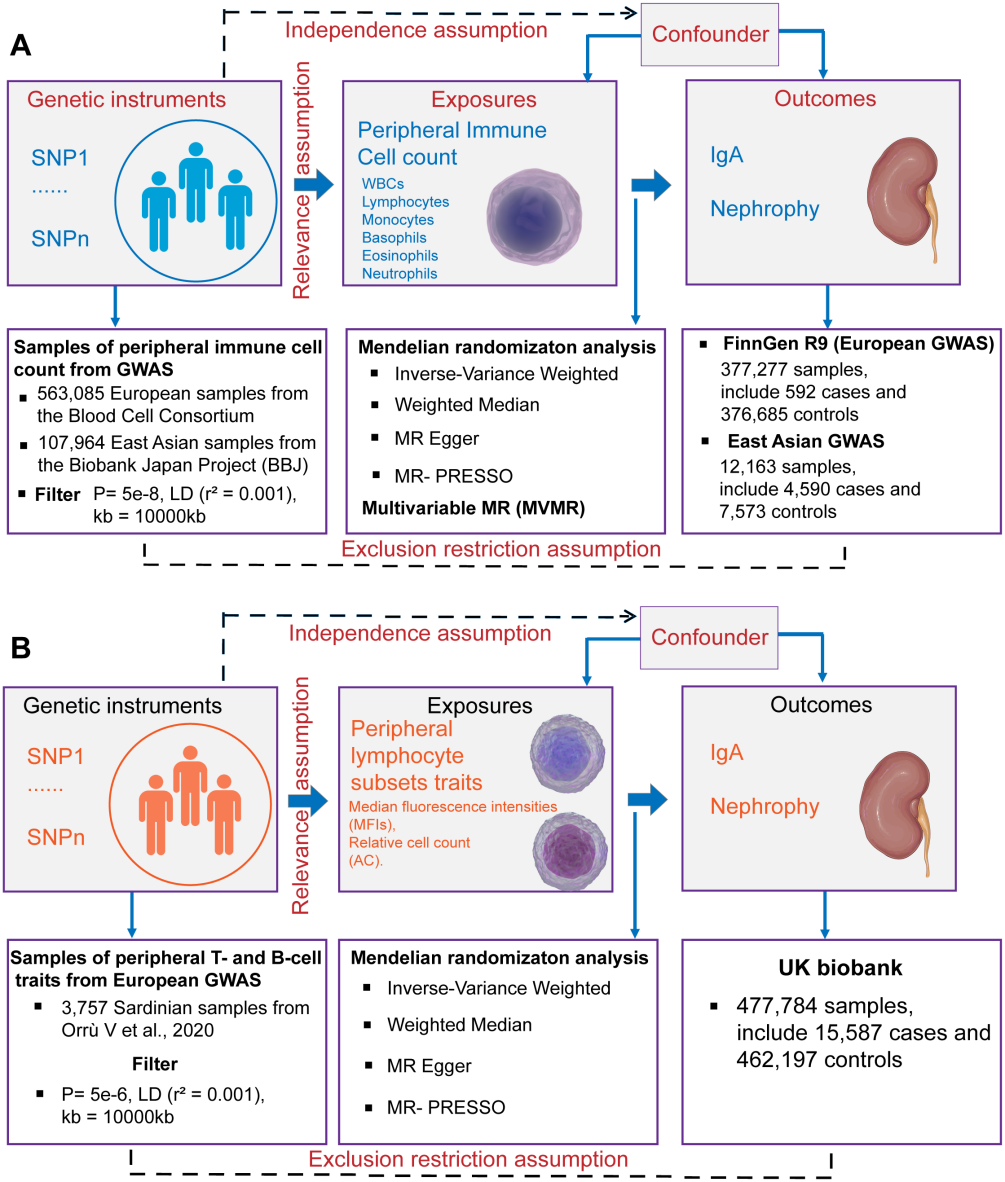
Schematics for Mendelian randomization concept and assumptions. **(A)** Schematic of MR analysis testing the effect of 6 immune cell subpopulation counts on IgAN. **(B)** Schematic of MR analysis testing the effect of peripheral immune cell subset characteristics on IgAN. WBCs, total white blood cells; GWAS, Genome-wide association studies; MR, Mendelian randomization; SNPs, single nucleotide polymorphisms; LD, linkage disequilibrium.

This study was conducted using the framework shown in **Figure 1**. Relevant single nucleotide polymorphisms (SNPs) were selected using quality control procedures based on separate, non-overlapping, published GWAS summaries. Different types of peripheral immune cells, including WBCs, lymphocytes, monocytes, basophils, eosinophils, and neutrophils, were considered. MR was used to assess the causal relationship between peripheral immune cells and IgAN with SNPs as IVs. For this, three assumptions must be met (25), as follows: (1) the genetic variants must be strongly associated with the exposure, (2) the SNPs must not be associated with a confounder of the risk factor-outcome association, and (3) the SNPs must only influence the outcome through the exposure of interest and not through any other pathway. A bidirectional and multivariable MR study was conducted in accordance with Strengthening the Reporting of Observational Studies in Epidemiology Using Mendelian Randomization (STROBE-MR) Statement (26).

### 2.2 GWAS data sources and data availability

Summary statistics for GWASs of peripheral immune cell counts, including total WBC, lymphocyte, monocyte, basophil, eosinophil, and neutrophil counts, were obtained from the Blood Cell Consortium (BCX), which includes data from 563,085 individuals of European ancestry (24). Individuals who are currently experiencing acute medical or surgical illness, end-stage renal disease, dialysis, or other similar conditions are not eligible to participate in the BCX Consortium (24). Additionally, data from the BioBank Japan Project (BBJ) were included, which encompasses individuals of East Asian ancestry (107,964 individuals for WBCs and 62,076 for other cell types) (27). The BBJ project is a multicenter, hospital-based registry cohort, for which approximately 200,000 individuals throughout Japan have been recruited (28). The final beta coefficient was found to be one standard deviation (SD) higher in cell counts with each additional effect allele. For the cellular subpopulation analysis, we selected the largest GWAS published to date for the immunophenotyping of peripheral blood cells. These GWAS summary statistics were obtained from 3,757 Sardinian samples containing 20,143,392 SNPs. Among these, 389 were median fluorescence intensities (MFIs) indicating the levels of surface antigens and 192 were relative cell counts (29).

The European GWAS analysis of IgAN (592 cases and 376,685 controls) was published on the FinnGen website. Larger summary-level statistical data for IgAN were extracted from a meta-analysis of GWASs in Europe, which included 15,587 cases and 462,197 controls from the UK Biobank and FinnGen (30). The UK Biobank project is a population-based cohort of 502,629 people with an average age of 56.8 years living in England, Scotland and Wales. The design of the cohort has been described previously (31). The FinnGen is a public–private partnership project involving 377,277 individuals (210,870 females and 166,407 males) living in Finland. Genotypic data were obtained from the Finnish Biobank and electronic health records from the National Register (32). In our study, we used the summary statistics of the FinnGen Release 9 data. The East Asian GWAS analysis of IgAN (4,590 kidney biopsy-diagnosed IgAN cases and 7,573 controls) was obtained from the six Asian cohorts (4). Summary statistics for European GWASs of membranous nephropathy (MN) (33) and diabetic nephropathy (DN) (30) were obtained from IEU Open GWAS project. The GWAS summary statistics for C-reactive protein (CRP) levels were obtained from a meta-analysis of 575,531 individuals of European ancestry from the GWAS Catalog (34). The European summary statistics are publicly available for download from the IEU Open GWAS project (https://gwas.mrcieu.ac.uk/), the FinnGen research project (https://www.finngen.fi/fi) and the GWAS Catalog (https://www.ebi.ac.uk/gwas/). The East Asian summary statistics are publicly available for download from JENGER (http://jenger.riken.jp/en/). All GWAS datasets and URLs are provided in the **Supplementary Tables S1**, **S2**.

### 2.3 Selection of IVs

SNPs associated with the counts of the six immune cell types were selected at a locus-wide significance threshold (*P* < 5×10^−8^) and defined as genetic instruments. For the GWAS summary statistics of Orrù et al. (29), who profiled 3,757 individuals via flow cytometry, SNPs associated with exposure were selected using the genome-wide significance levels (*P* < 5×10^−5^). This was necessary because the number of SNPs included under the locus-wide significance threshold (*P* < 5×10^−8^) was too limited, potentially resulting in the loss of meaningful findings. To ensure a close association with IgAN, we selected SNPs that were significantly associated with kidney diseases with a *P*-value of 5×10^−8^ as IVs. To eliminate linkage disequilibrium and maintain the independence of the selected IVs, PLINK software (version v1.92) was used to prune these SNPs. The clumping distance was set to 10,000 kb and r^2^ was set to less than 0.001, based on the 1000 Genomes Project (35). The validity of the IVs was assessed using the F statistic (36). The F-values were calculated using the following formula: F = (R^2^ × (N − 2))/(1 − R^2^), where N represents the sample size and R^2^ is the proportion of phenotypic variance explained by genetic instruments (37). Only IVs with F-statistics greater than 10 were included in the analysis to avoid weak instrumental bias. A Steiger filter was used to eliminate instruments with an outcome larger than the exposure R^2^ (38). After harmonization, palindromic SNPs with a minor allele frequency less than 0.3 in the exposure GWAS, which could exhibit pleiotropic effects, were removed from the analyses. Finally, SNPs that met the rigorous screening criteria were incorporated into bidirectional MR analysis. We obtained a total of 2–465 independent SNPs in SVMR, which could explain an average of 4.99% (range, 0.04–13.4%) of the variance in each exposure. Further details on the IVs used in this study are provided in **Supplementary Table S3**.

### 2.4 Bidirectional and multivariate MR analysis

Single-variable MR (SVMR) and reverse MR analyses were performed to examine the bidirectional relationships between different immune cell counts and traits in the peripheral blood and IgAN. Additionally, we also examined the potential causal relationship between distinct immune cell counts and MN and DN through the use of SVMR methods. We employed four different methods for SVMR analysis, including the inverse variance weighted (IVW) (39), weighted median estimator (40), MR Egger regression (MR-Egger) (41), and MR pleiotropy residual sum and outlier (MR-PRESSO) methods (42). To assess the independent influence of immune cell types on the IgAN risk, multivariable MR (MVMR) was performed (43), considering the interplay between different immune cell types and CRP levels. The MVMR analysis was performed using the IVW method. The direct effect of each immune subtype was estimated by adding the CRP levels to the MVMR model. The IVW method was considered the most efficient when the genetic variants were independent of each other, and there was no evidence of horizontal pleiotropy in the selected IVs (Egger intercept and MR-PRESSO global test *P*-value > 0.05). MR estimates were reported as odds ratios (ORs) with 95% confidence intervals (95% CIs), which represent the risk of the kidney diseases observed with each unit increase in the inverse normalized immune cell count or immune cell traits.

### 2.5 Sensitivity and statistical analyses

We primarily used the IVW method to assess the relationship between exposures and outcomes (39). Subsequently, a series of comprehensive sensitivity analyses were conducted to evaluate the robustness of the IVW results against potential violations of MR assumptions. These included the weighted median (40), MR-Egger (41), MR-PRESSO (42) and MVMR analyses (43). Cochran’s Q test was used to test for heterogeneity. The potential influence of heterogeneity on the causal outcome was disregarded at *P* > 0.05. When heterogeneity was significant (*P* < 0.05), the IVW-RE model was used to reduce its impact on the causal effects (44, 45). The MR-PRESSO method was used to identify potential outliers (42). The presence of horizontal pleiotropy was evaluated using the MR-Egger regression intercept method (46) and MR-PRESSO global test (42). Funnel plots were used to visualize symmetry and estimate the effect. To correct for multiple testing across analyses, we implemented a Bonferroni correction to establish *P*-value thresholds for strong evidence (false discovery rate-adjusted *P* = 0.05/number of risk factors in the models) and suggestive evidence (*P_FDR_* < *P* < 0.05) for the reported associations. A causal relationship was deemed to exist when the following criteria were met: the results of IVW and at least one sensitivity analysis were statistically significant (*P* < 0.05), all sensitivity analyses yielded estimates that were in agreement, there was no evidence of horizontal pleiotropy and heterogeneity, defined as *P*> 0.05 for the MR-Egger intercept test, MR-PRESSO global pleiotropy test and Cochran’s Q statistic. MR analyses were performed using the Two-Sample MR (version 0.5.7), MR-PRESSO (version 1.0), and MVMR (version 0.5.7) packages in R (version 0.5.7).

## 3 Results

### 3.1 Univariable MR analysis (SVMR) of the association between peripheral immune cell counts and the IgAN risk

**Figure 2:**
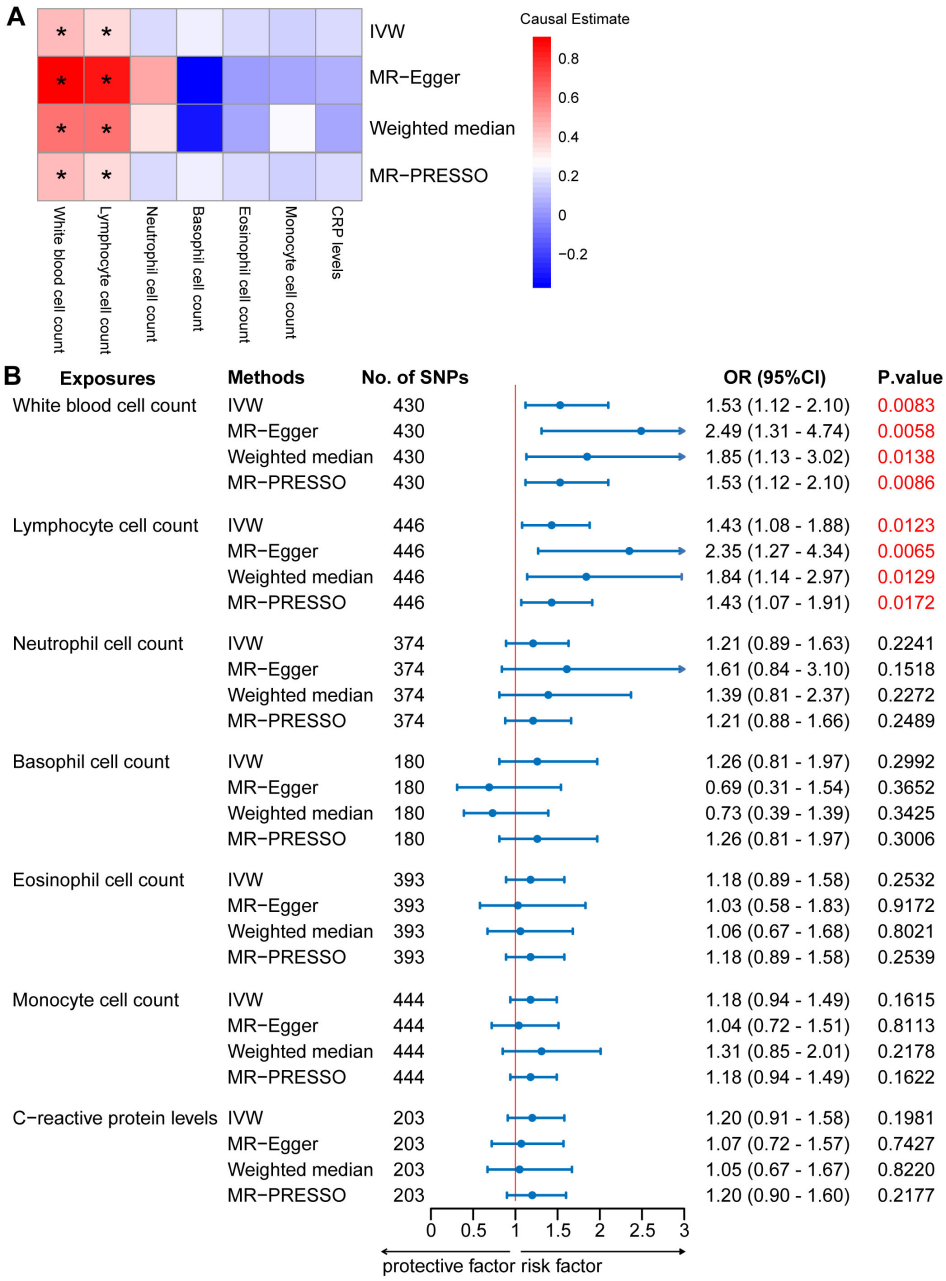
Mendelian randomization (MR) analyses testing the effects of 6 immune cell subpopulation counts and CRP levels on IgAN. **(A)** Results obtained using four MR approaches are presented as a heat map showing causal estimates (beta). *P <0.05 for a particular MR methods. **(B)** MR estimates of the association between immune cell subpopulation counts and CRP levels and risk of IgAN. IVW, Inverse Variance Weighted; MR-Egger, Mendelian randomization-Egger; MR-PRESSO, Mendelian Randomization Pleiotropy Residual Sum and Outlier; OR, Odds Ratio; CI, Confidence Interval.

In the two-sample MR analysis, we primarily used the IVW method to measure the relationship between IgAN and peripheral immune cell counts. The MR analysis of European GWAS revealed a strong association between higher total WBC count and increased susceptibility to IgAN (OR: 1.53; 95% CI: 1.12–2.10; *P* = 0.0083) based on IVW analysis, even after correcting for multiple testing (*P* ≤ 0.05/6 for the six immune cell counts; **Figure 2**, **Supplementary Table S4**). Sensitivity analysis results showed a consistent trend. Upon visual inspection of the funnel plots, no obvious directional pleiotropy was detected (**Supplementary Figure S1**). However, the MR-PRESSO global pleiotropy test revealed the presence of overall horizontal pleiotropy among all genetic instruments (P-values <0.05) (**Supplementary Table S4**). Based on the heterogeneity test, there was significant heterogeneity (Cochran’s Q *P*-values < 0.05).

Among the five main subtypes of WBCs, evidence was found for the genetically predicted lymphocyte counts (OR: 1.43; 95% CI: 1.08–1.88; *P* = 0.0123), with an effect estimate that was consistent with an increased risk of IgAN (**Figure 2**, **Supplementary Table S4**). The MR-Egger analysis revealed that a one-unit increase in the lymphocyte count was causally associated with a 135% relative increase in the IgAN risk (OR: 2.35; 95% CI: 1.27–4.34; *P* = 0.0065). The estimates were consistent between the weighted median and MR-PRESSO methods. Horizontal pleiotropy was not indicated based on the MR-Egger intercept test and MR-PRESSO global pleiotropy test. There was no significant heterogeneity based on the heterogeneity test (Cochran’s Q *P*-value > 0.05) (**Supplementary Table S5**). We also investigated the causal association between the levels of CRP, a marker of systemic inflammation, and IgAN levels, as circulating immune cell abundance is particularly susceptible to changes associated with infection or injury. There was no evidence of an association between CRP and the risk of IgAN (OR: 1.20; 95% CI: 0.91–1.58; *P* = 0.1981; **Figure 2**, **Supplementary Table S4**). Reverse causation analyses were also performed, but no meaningful insights were gained from the analyses of IgAN and immune cell counts, including WBC, lymphocyte, basophil, eosinophil, neutrophil, and monocyte counts (OR: 1.00) (**Supplementary Table S6**).

The MR analysis of East Asian GWAS showed a median association between elevated monocyte counts and heightened susceptibility to IgAN (OR: 1.47; 95% CI: 1.10–1.98; *P* = 0.0104) based on IVW analysis (**Supplementary Figure S2**, **Table S4**). This estimate was consistent with that obtained using the MR-PRESSO method. Although no horizontal pleiotropy was detected by the MR-Egger intercept test, horizontal pleiotropy was identified by the MR-PRESSO global pleiotropy test. The heterogeneity test indicated significant heterogeneity (**Supplementary Figure S3**, **Table S5**).

### 3.2 MVMR analysis of association between peripheral immune cell counts and IgAN risk

The MVMR-IVW analysis was used assessed the genetic liability association between peripheral immune cell counts and IgAN, adjusting for the effects of CRP levels. The lymphocyte cell count retained a positive relationship with IgAN (OR:1.44; 95% CI: 1.05–1.96; *P* = 0.0210; **Supplementary Table S7**), which was consistent with the findings of SVMR. The sensitivity analyses comprising MVMR-Egger and MV-MR-PRESSO methods produced effect estimates consistent with those observed with MVMR-IVW. No heterogeneity or horizontal pleiotropy was detected using the MV-MR Egger heterogeneity test and MR-PRESSO Global pleiotropy test (*P*-values > 0.05; **Supplementary Table S7**).

### 3.3 MR analysis of the association between lymphocyte subset traits and IgAN risk

**Figure 3:**
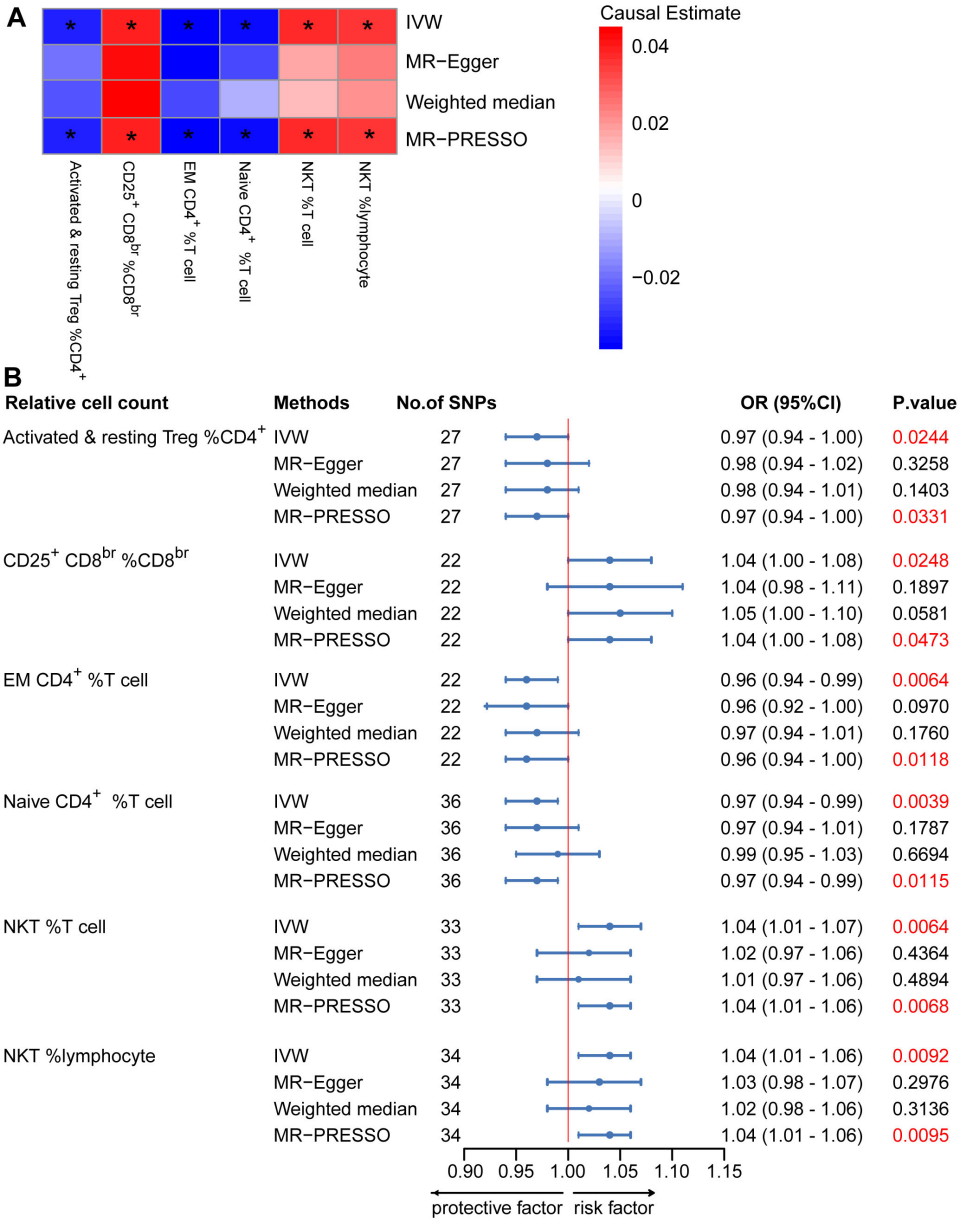
Mendelian randomization (MR) analyses testing the effects of the relative count of T-cell subpopulation on IgAN. **(A)** Results obtained using four MR approaches are presented as a heat map showing causal estimates (beta). *P <0.05 for a given MR method. **(B)** MR estimates of the association between T-cell subpopulation relative counts and risk of IgAN. IVW, Inverse Variance Weighted; MR-Egger, Mendelian randomization-Egger; MR-PRESSO, Mendelian Randomization Pleiotropy Residual Sum and Outlier; OR, Odds Ratio; CI, Confidence Interval.

**Figure 4:**
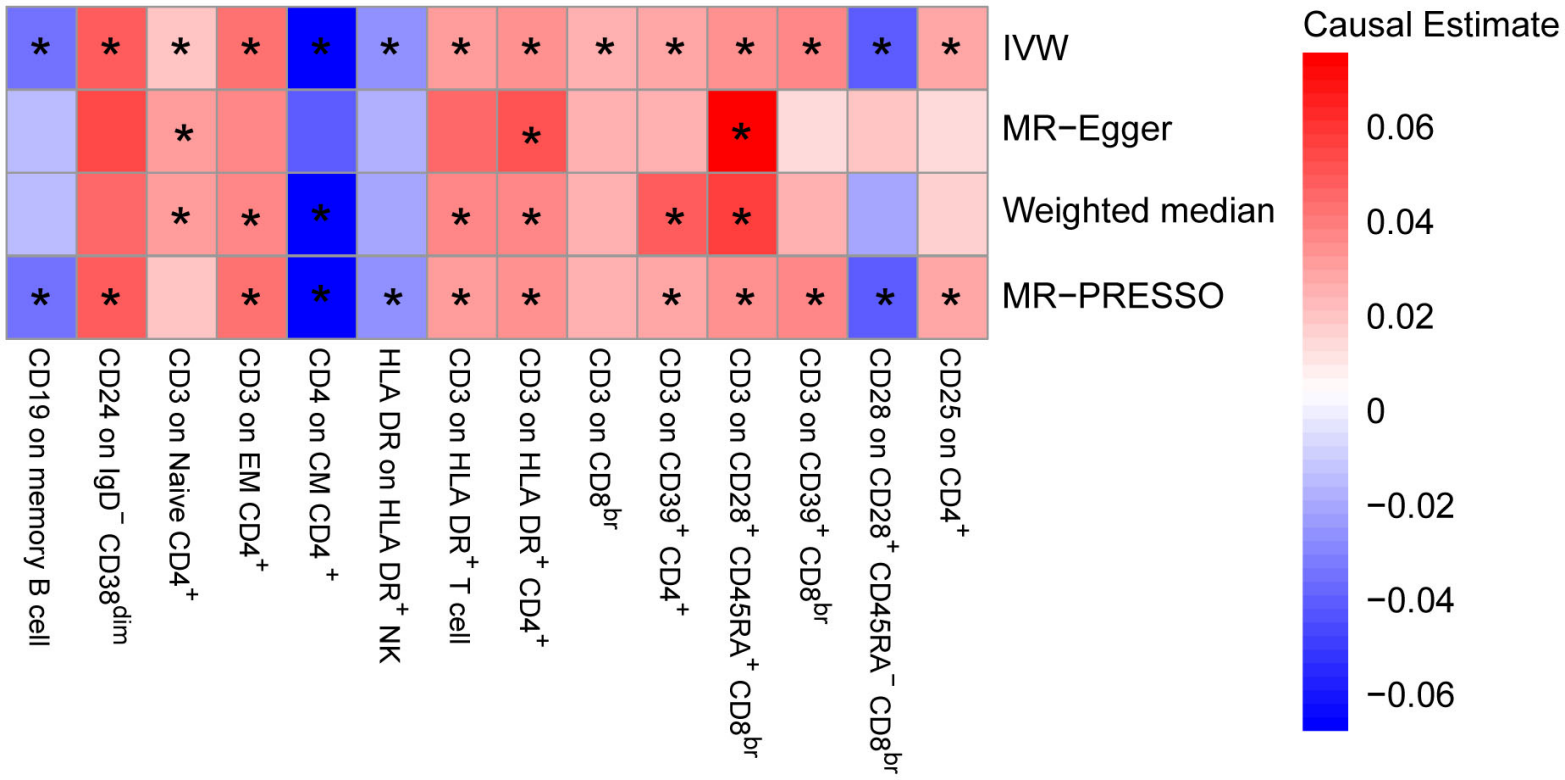
Mendelian randomization (MR) analyses testing the effects of lymphocyte subset immune trait (MFIs) on IgAN. Results obtained using four MR approaches are presented as a heat map showing causal estimates (beta). *P <0.05 for a particular MR method. IVW, Inverse Variance Weighted; MR-Egger, Mendelian randomization-Egger; MR-PRESSO, Mendelian Randomization Pleiotropy Residual Sum and Outlier; MFIs, Median Fluorescence Intensities.

**Figure 5:**
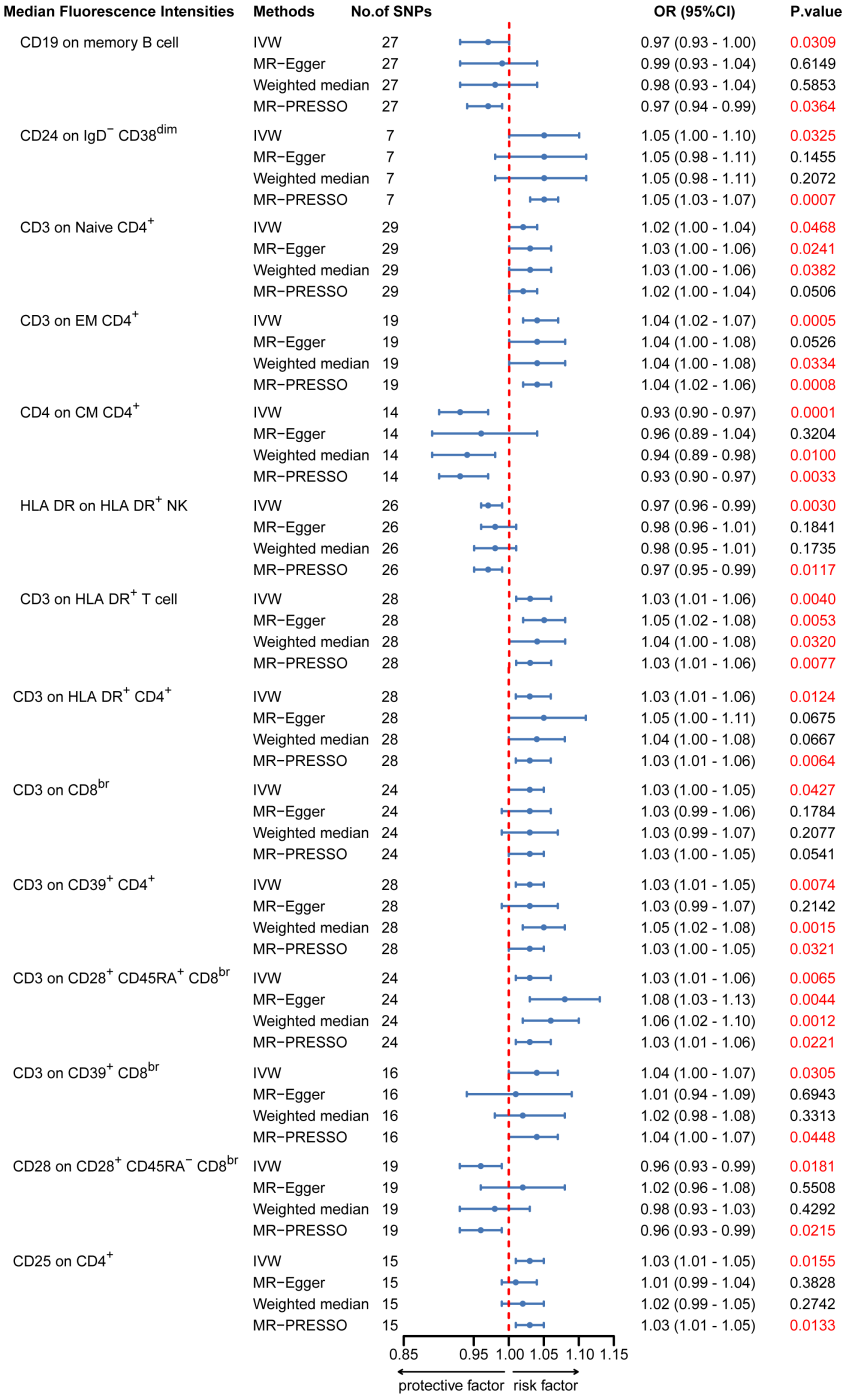
MR estimates of the association between lymphocyte subset immune trait (MFIs) and risk of IgAN. IVW, Inverse Variance Weighted; MR-Egger, Mendelian randomization-Egger; MR-PRESSO, Mendelian Randomization Pleiotropy Residual Sum and Outlier; OR, Odds Ratio; CI, Confidence Interval; MFIs, Median Fluorescence Intensities.

To provide a more complete understanding of the causal relationship between immune indicators and IgAN, we selected the largest GWAS published to date for peripheral blood immunophenotyping, as described in Orrù’s study (29). This study included 389 MFI traits reflecting the levels of surface antigens and 192 relative cell count traits. The analysis was primarily performed to measure the causal effects of T and B cell counts on IgAN risk, as these are the two major lymphocyte subpopulations implicated in the disease. The IVW method revealed that an increase in several relative T cell subset counts was associated with a higher risk of IgAN. Specifically, CD25^+^ CD8^br^% CD8^br^ (OR: 1.04; 95% CI: 1.00–1.08; *P* = 0.0248), NKT %T cell (OR: 1.04; 95% CI: 1.01–1.07; *P* = 0.0064), and NKT % lymphocytes (OR, 1.04; 95% CI, 1.01–1.06; *P* = 0.0092) were found to be significant. This finding was supported by two other MR methods (**Figure 3**; **Supplementary Table S4**).We also identified 14 pairs of MFIs in lymphocyte subsets and IgAN that reached a suggestive association (*P* < 0.05) based on an IVW analysis (**Figures 4**, **5**; **Supplementary Table S4**). The IVW results revealed two immune traits associated with IgAN, namely CD19 on memory B cells (OR: 0.97: 95% CI: 0.93–1.00: *P* = 0.0309) and CD24 on IgD^–^ CD38^dim^ cells (OR: 1.05; 95% CI: 1.00–1.10; *P* = 0.0325). In particular, genetic evidence suggested that an increase in CD3 on several T cell panels is associated with a higher risk of IgAN (e.g., CD3 on naive CD4^+^; CD3 on EM CD4^+^; CD3 on naive CD4^+^; CD3 on HLA DR^+^ CD4^+^; CD3 on CD8^br^; CD3 on CD39^+^ CD4^+^; CD3 on CD28^+^ CD45RA^+^ CD8^br^; CD3 on CD39^+^ CD8^br^). Conversely, the evidence across analyses suggested a potential protective effect of an increase in three T cell and NK cell traits (CD4 on CM CD4^+^ T cells; HLADR on HLA DR^+^ NK cells; CD28 on CD28^+^ CD45RA^−^CD8^br^ Treg cells). Additionally, we conducted reverse causation analyses and determined that genetic predisposition to IgAN, as an exposure, did not causally affect the aforementioned lymphocyte subpopulations in bidirectional MR analyses (**Supplementary Table S6**).

### 3.4 SVMR analysis of the association between peripheral immune cell counts and other kidney diseases risk

To ascertain whether peripheral immune cell counts are a risk factor for MN and DN, a SVMR analysis was performed. While the IVW analysis results indicated an association between higher total WBC, lymphocyte, and neutrophil counts and decreased susceptibility to MN, there were significant horizontal pleiotropy and heterogeneity (**Supplementary Figures S4**, **S5**, **Tables S4**, **S5**). Consequently, there is currently no compelling evidence to support a causal relationship between total WBCs, lymphocyte and neutrophil counts and MN. Similarly, no meaningful findings were gained from the analyses of DN and immune cell counts, including WBC, lymphocyte, basophil, eosinophil, neutrophil, and monocyte counts (**Supplementary Figures S6**, **S7**, **Tables S4**, **S5**).

## 4 Discussion

Although the immune system plays a significant role in the pathogenesis of IgAN, its contribution to IgAN remains unclear. To the best of our knowledge, this is the first MR analysis to investigate the potential causal relationship between peripheral immune cells counts and traits and IgAN. Using MVMR, we evaluated the independent causal effects of six immune cell counts while adjusting for CRP levels. The main result of this study suggest that a high lymphocyte count is a causal risk factor for IgAN in Europe. Furthermore, CD3, expressed in eight subsets of T cells, consistently showed a positive correlation with IgAN.

The most prominent clinical signs of IgAN are hematuria, proteinuria, and progressive renal failure. The 2021 Guidelines for Managing Blood Pressure in Chronic Kidney Disease Patients with IgAN recommend using renin–angiotensin–aldosterone system blockers as conservative supportive therapy for IgAN to manage blood pressure and reduce proteinuria (47). This finding is particularly important, given that a recent study provided evidence of a causal relationship between circulating WBCs and the risk of high blood pressure (48). Previous studies on IgAN have identified independent disease-associated loci involving mucosal innate immunity using a GWAS approach (4–7). Kiryluk et al. confirmed the complex polygenic architecture of IgAN, with extrarenal cell types, including immune and gastrointestinal mucosal cells, having a probable causal role (4). Our study proposes a possible mechanism for the increased risk of IgAN associated with lymphocytes, through elevated blood pressure parameters.

A substantial body of research has demonstrated a causal relationship between peripheral immune cells and various diseases. For instance, genetic variants associated with eosinophil counts and asthma (49), circulating WBC counts and colorectal cancer (50) and neutrophil counts and atherosclerotic cardiovascular disease had been shown to be causally related (51). Our MR analysis suggests a different causal relationship between peripheral immune cells and IgAN in European and East Asian individuals. Genetic instruments (range, 7–30 independent IVs) for peripheral immune cells were selected from a genome-wide pQTL mapping GWAS in 107,964 (WBCs) and 62,076 (other five cell subtypes) East Asian individuals. In European GWAS, however, 203–446 total independent IVs are available for further analysis. Given the limited number of genetic instruments available as proxies for the peripheral immune cell counts in East Asians, these results likely reflect limited statistical power. It is well known that IgAN develops as a result of a combination of environmental and multifactorial genetic background factors. Sporadic IgAN, racial differences and regional differences have been reported (52). Thus, this could be the reason for the differences in MR results between European and East Asian individuals. Given that horizontal pleiotropy was detected by the MR-PRESSO global pleiotropy test and may result in distortion of causal estimates in MR, there is currently no compelling evidence to support a causal relationship between monocyte counts and IgAN in East Asian populations.

Clinical studies have shown that patients with IgAN exhibit higher levels of B cells, plasma cells and monocytes when compared to healthy controls. These cells may play a role in the development of IgAN (17, 20). D’Amico and his colleagues showed that patients with idiopathic IgAN exhibit a significant increase in the interstitial accumulation of monocytes and T lymphocytes (53). Consistent with this, our results showed a median association between elevated peripheral lymphocyte counts and heightened susceptibility to IgAN in Europeans. However, our MR analysis did not find a significant causal association between six peripheral blood immune cells and other kidney diseases (including MN and DN). There are differences in the pathogenesis of the three kidney diseases. MN is a pathological glomerular disorder characterized by thickening of the glomerular capillary walls and deposition of immune complexes consisting of immunoglobulin G (IgG), complement components, and relevant antigens (54). DN is a common microvascular complication in patients with diabetes worldwide. It’s pathophysiology involves the glomeruli and renal tubules. There is increasing evidence that inflammatory factors are critical in the development of DN (55).

Immunosuppression trials have shown considerable benefits for IgAN. Although lymphocytes constitute less than 1% of peripheral WBCs in humans, they play a crucial role in mediating immune mechanisms. Given that the regulatory effects of variants are often dependent on cellular subtypes, deciphering their pathologic roles will require an analysis of the disease-relevant cell types (56, 57). This study focused on specific lymphocyte subtypes and their roles in IgAN. T lymphocyte imbalances comprise a crucial immune mechanism in IgAN. Our MR analysis revealed a significant causal relationship between relative cell count of CD25^+^CD8^br^ %CD8^br^, NKT %T cell and NKT % lymphocyte and IgAN. CD25 is expressed in the T cell population, and it increases the receptor affinity for interleukin-2 (IL-2) cytokine. IL-2 and its receptor, which contains CD25, interact on the antigen-specific activated regulatory T cells, cytotoxic lymphocytes and T helper cells, resulting in cell growth and differentiation (58). In line with this, a small clinical study showed that serum IL-2 levels are elevated in IgAN (59). Natural killer T (NKT) cells make up a small proportion of blood T cells in healthy individuals and, based on previous studies, appear to have a protective effect on IgAN (60). In contrast, our results suggest that the relative NKT cell count increases the risk of developing IgAN. Given the limited number of SNPs available as proxies for the immune cell traits of NKT cells, our results likely reflect the limited statistical power. Additionally, our genetic evidence suggests that an increase in CD3, a T cell marker that indicates interstitial inflammation, is associated with a higher risk of IgAN. Through multivariate analysis, an observational study discovered that CD3 scores are independently associated with progressive IgAN (61). Further, in a study of markers of tubulointerstitial leukocyte and cytokine infiltration, Myllymaki et al. found that CD3(+) cell infiltration, indicative of T cells, was most effective, among tubulointerstitial injury markers, in predicting disease progression (62, 63). Although a positive correlation between CD3 (+) T cells and IgAN has been observed in epidemiological studies, our study corroborated these correlations based on genetics, thus providing empirical support for the hypothesis that IgAN might be caused by the dysfunction of a relatively minor proportion of T cells, which is associated with more prominent effects. Despite the absence of a causal relationship between B cell subtype counts and IgAN in our MR analysis, our results revealed that some disease-associated immune features of B cells (e.g., CD19 on memory B cells, CD24 on IgD−CD38^dim^ cells) exhibit interactions with IgAN. Interestingly, CD19 expression on B cells is involved in the regulation of B cell receptor (BCR) signaling, B cell development and humoral immunity (64). CD19 (+) memory B cells and CD24 (+) IgD^–^CD38^dim^ cells comprise an inactivated cell subtype, and upon antigen-specific stimulation, these B cells can differentiate into plasma cells and plasmablasts. Previous studies have demonstrated that CD24 polymorphisms are associated with an increased risk of autoimmune diseases, including systemic lupus erythematosus, multiple sclerosis, and rheumatoid arthritis (65–67). Consistent with this, our findings suggest an increased risk for CD24 on IgD^–^CD38^dim^ cells in IgAN. The activation of B lymphocytes is directly associated with early IgAN stages (68), and there is strong evidence to suggest that this disease is mediated by the glomerular deposition of Gd-IgA1 (13, 69, 70). IgA is typically produced by the mucosa-associated lymphoid tissue, and it is found in various areas of the body (71, 72). Pathogenic bacteria in the mucosa-associated lymphoid tissue promote the maturation and differentiation of immature B lymphocytes through both T cell-independent and T cell-dependent pathways. This results in a class switch from IgM to IgA1 and promotes IgA overproduction (73–75). Nakayamada et al. also showed that inhibiting B lymphocyte activation and lowering IgA levels represent promising strategies for investigating IgAN therapeutics (76). Further experimental evidence is required to clarify the potential roles of lymphocytes in IgAN pathogenesis.

Our study has several strengths. First, the large sample size in the GWAS increased the measurement precision in the first part of the MR analyses. Additionally, the MVMR models have significant strengths, and the rigorous screening of IVs greatly improved the reliability of our results. Although no single method can entirely protect the findings of the MR analyses from the influence of potential pleiotropic effects, a consistent effect estimate derived from multiple sensitivity analyses adds confidence to the plausibility of a true underlying causal effect. By comparing the results of multiple sensitivity analyses, we minimized potential bias resulting from pleiotropic effects. However, causal inferences must be carefully interpreted. Second, F-statistics greater than 10 suggest that our results are unlikely to suffer from weak instrumental bias. Third, Lymphocytes comprise a diverse population of cells with distinct phenotypic and functional properties. In the last part of the MR analyses, we examined specific subsets of circulating lymphocytes through fluorescence-activated cell sorting. We further revealed that changes in the circulating lymphocyte components might affect the risk of IgAN. Fourth, research has indicated that inflammation could be a risk factor for IgAN development. Our study found that adding CRP levels (a marker of systemic inflammation) to the MVMR analysis did not result in a significant change in statistical power. These analyses suggest that inflammation has no direct independent effect on the risk of developing IgAN.

However, this study also has some limitations. First, given the relatively modest scale of the lymphocyte-phenotyping GWAS and East Asians GWAS, the final part of our MR analyses was limited by the availability of variants that could be effectively used as IVs. Thus, these results likely reflect limited statistical power. Although relaxing the association threshold to a *P*-value <5 x 10^−5^, as adopted by Orrù et al. in their MR analyses (29), would provide more IVs, it would also compromise the first MR assumption. In addition, with the increasing scale of phenotyping GWASs in the future, we expect a greater number of SNPs surpassing the GWAS significance threshold that can be reliably used as IVs for an MR analysis. Second, the study focused on populations of European and East Asian descent for genetic homogeneity; therefore, the generalizability of the findings to other ethnic groups is uncertain. Further genetic profiling of diverse ethnic groups is thus required. Third, in the present study, we analyzed causal associations between lymphocyte counts and a select few kidney diseases. Our findings do not permit us to conclude whether lymphocyte counts are associated with other forms of kidney disease, due to the lack of publicly available GWAS data. Fourth, given that no MR analysis has been performed to entirely exclude the effects of pleiotropy, while the sensitivity analyses have failed to identify any evidence of horizontal pleiotropy, there is still the possibility of an association that could be influenced by other causal pathways. Nevertheless, the fulfillment of the established criteria for a causal relationship should, in theory, result in minimal confusion and bias. Fifth, the present study analyzed data from the BCX Consortium and BBJ, wherein the mean peripheral immune cell counts were within the normal range (27, 77). This assumes that the lymphocyte counts were constant and did not allow us to establish a relationship between the trend in immune cell counts and IgAN risk. However, studying the relationship between peripheral blood cell counts and disease development is worthwhile. Finally, the genetic instruments used in the MR analysis were proxied for lifetime variations in lymphocyte counts. Therefore, the size of the estimate should be interpreted cautiously, as it is unlikely to reflect the effect of the modifying exposure at a given time point. Thus, these results cannot be used to infer the effects of significant changes in IgAN over a short period. In conclusion, our findings indicate that genetically determined higher lymphocyte counts are associated with IgAN, suggesting that a high lymphocyte count is a causal risk factor for IgAN. These findings may have implications for the treatment of immunosuppression and their utility as a biomarker in disease risk assessment.

## Data Availability

The original contributions presented in the study are included in the article/**Supplementary Material**. Further inquiries can be directed to the corresponding author/s.
