# Supplementary material for: Causal association between peripheral immune cells and IgA nephropathy: a Mendelian randomization study
Source: Front Immunol. 2024 Aug 16;15:1371662. doi: 10.3389/fimmu.2024.1371662 (PMC11361932; doi:10.3389/fimmu.2024.1371662)
Supplement: Supplementary file 2 [file DataSheet1.docx]

Supplementary Material

# Supplementary Figures and Tables

## Supplementary Figures

Supplementary Figure S1. Funnel plot to assess heterogeneity (IgAN).

Supplementary Figure S2. Scatter plot of SNPs associated with peripheral immune cells count (East Asian) and the risk of IgAN (East Asian).

Supplementary Figure S3. Funnel plot to assess heterogeneity (East Asian).

Supplementary Figure S4. Scatter plot of SNPs associated with peripheral immune cells count and the risk of membranous nephropathy (MN).

Supplementary Figure S5. Funnel plot to assess heterogeneity (Membranous nephropathy, MN).

Supplementary Figure S6. Scatter plot of SNPs associated with peripheral immune cells count and the risk of diabetic nephropathy (DN).

Supplementary Figure S7. Funnel plot to assess heterogeneity (Diabetic nephropathy, DN).

**1.2 Supplementary Tables**

Supplementary Table S1. Information of the Exposure Datasets.

Supplementary Table S2. Information of the Outcomes Datasets.

Supplementary Table S3. Detailed information of instrumental variables utilized in the Mendelian Randomization analysis.

Supplementary Table S4. Sensitivity analysis of Mendelian randomization estimates for peripheral immune cells with different nephropathy using different methods.

Supplementary Table S5.MR estimates from each method of assessing the causal effect of exposure on the risk of outcome (Heterogeneity Test) .

Supplementary Table S6. Reverse MR analyses evaluating the causal effects of IgAN on peripheral immune cells using different methods.

Supplementary Table S7. Sensitivity analysis of multivariate Mendelian randomization (MVMR) estimates for peripheral immune cells with IgAN using different methods.


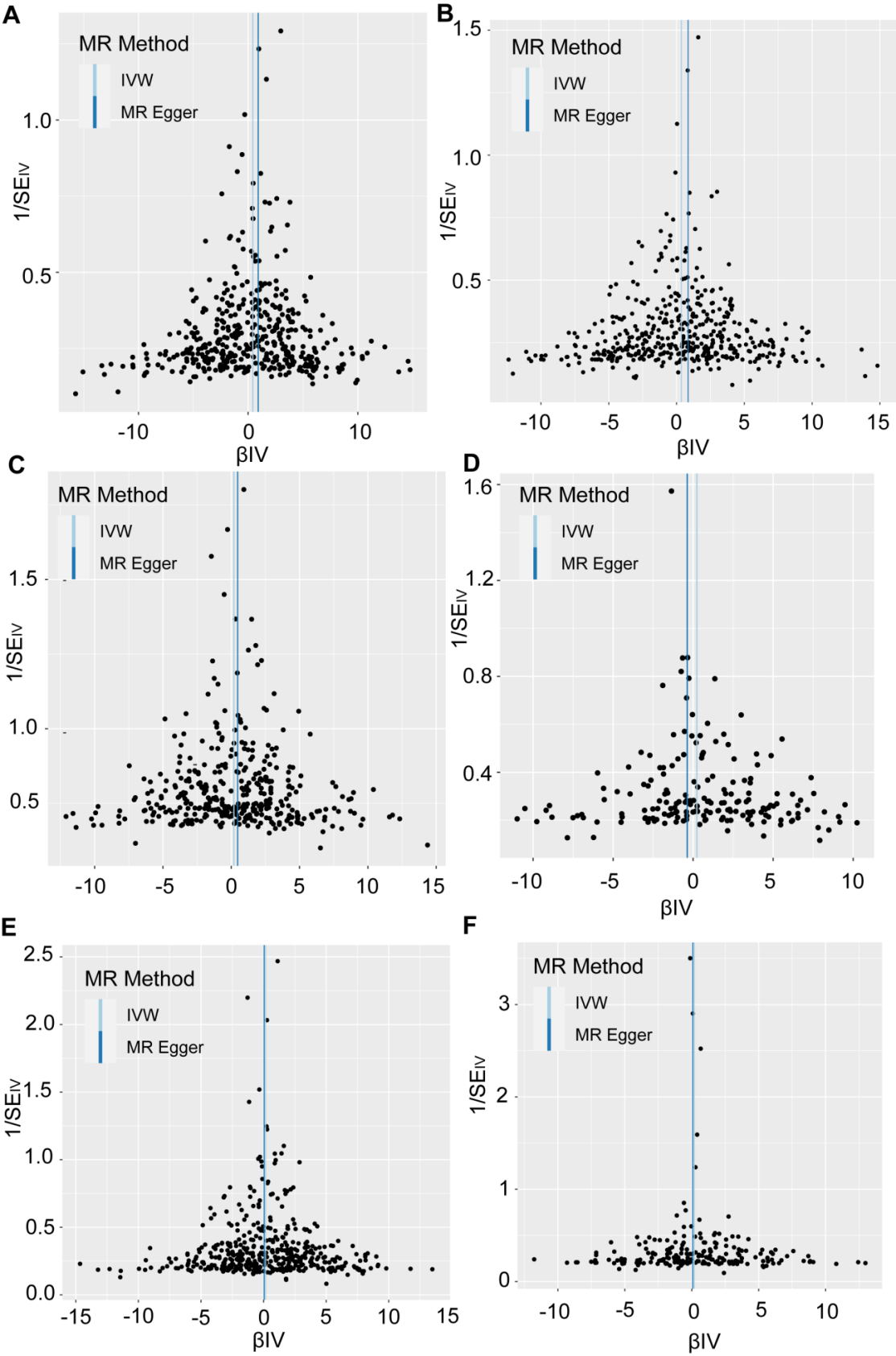


**Supplementary Figure S1.** **Funnel plot to assess heterogeneity (IgAN).** Funnel plot of SNP for MR analysis of exposures on outcomes. The light blue line represents the inverse‐variance weighted estimate. The dark blue line represents the MR-Egger estimate. (A) MR estimates for total white blood cell count on IgAN. (B) MR estimates for lymphocyte cell count on IgAN. (C) MR estimates for neutrophil cell count on IgAN. (D) MR estimates for basophil cell count on IgAN. (E) MR estimates for monocyte cell count on IgAN. (F) MR estimates for C-reactive protein levels on IgAN.


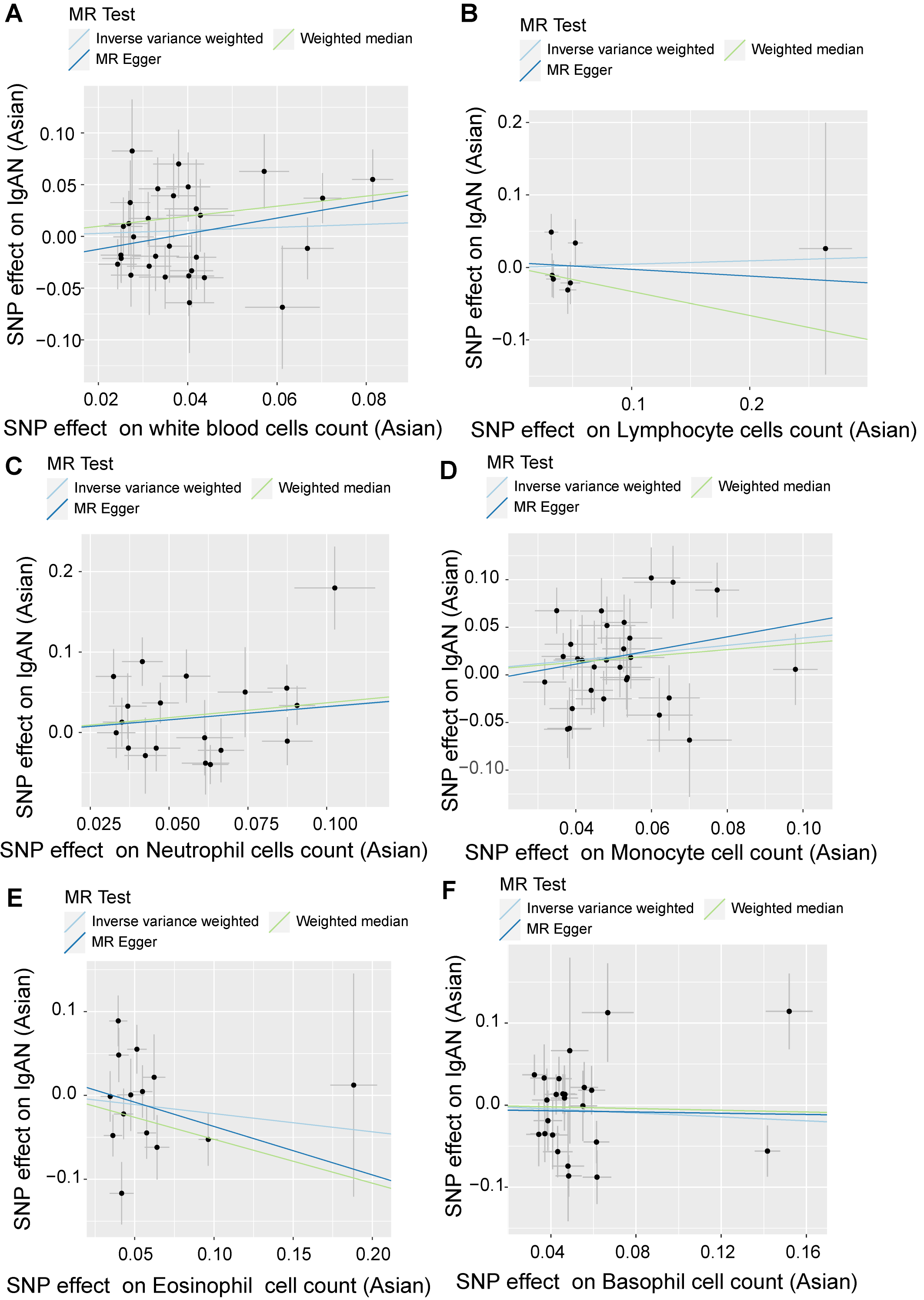


**Supplementary Figure S2. Scatter plot of SNPs associated with peripheral immune cells count (East Asian) and the risk of IgAN (East Asian)**. The plot presents the effect sizes of the SNP-peripheral immune cells count association (x-axis, SD units) and the SNP-IgAN (Asian) association (y-axis, log (OR)) with 95% conffdence intervals. The regression slopes of the lines correspond to causal estimates using the three Mendelian randomization (MR) methods (the Inverse variance weighted method, weighted median estimator, and MR-Egger). (A) White blood cell count on IgAN (East Asian); (B) Lymphocyte cell count on IgAN (East Asian). (C) Neutrophil cell count on IgAN (East Asian). (D) Monocyte cell count on IgAN (East Asian). (E) Eosinophil cell count on IgAN (East Asian). (F) Basophil cell count on IgAN (East Asian).


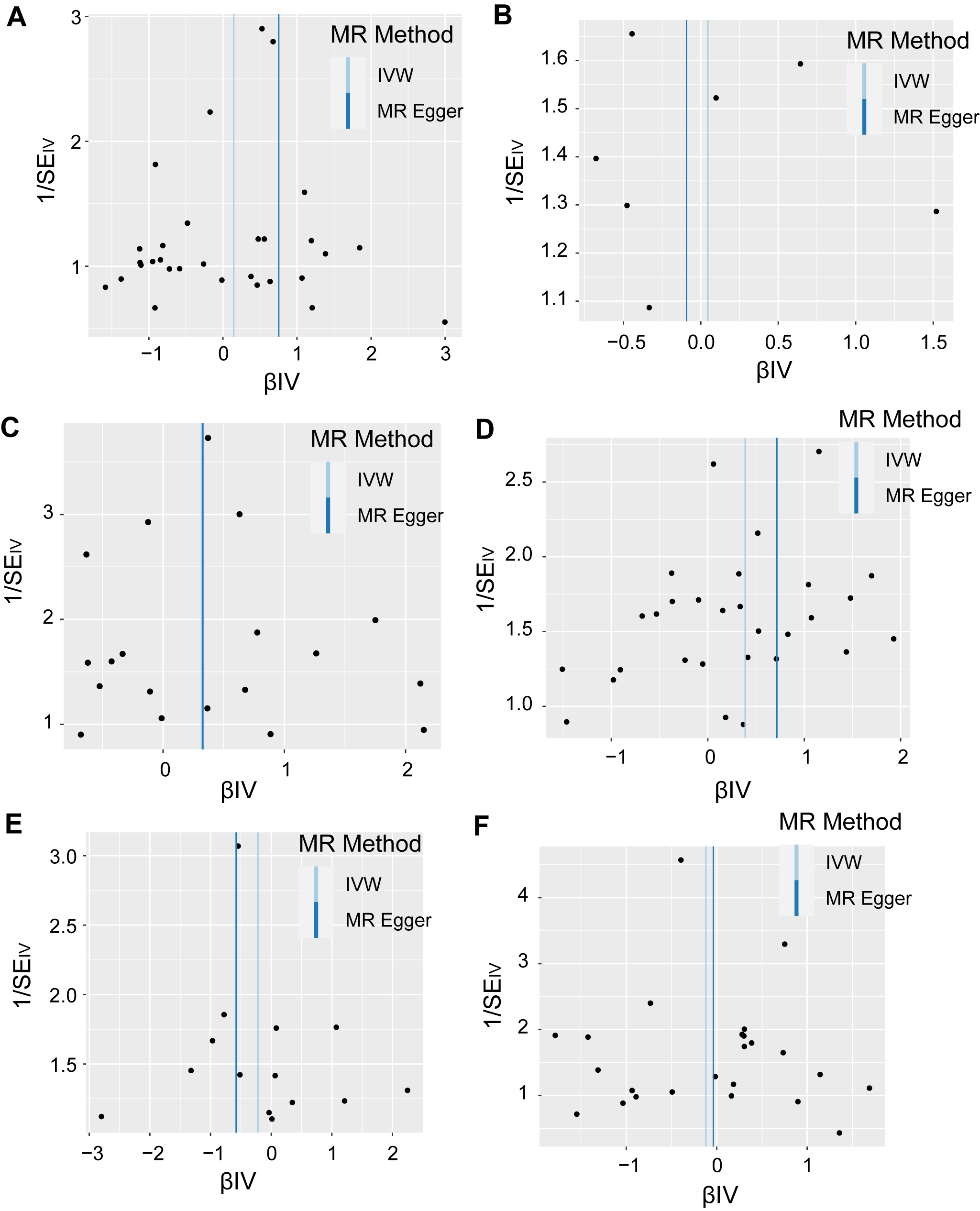


**Supplementary Figure S3.** **Funnel plot to assess heterogeneity (East Asian).** Funnel plot of SNP for MR analysis of exposures on outcomes. The light blue line represents the inverse‐variance weighted estimate. The dark blue line represents the MR-Egger estimate. (A) MR estimates for white blood cell count on IgAN (East Asian). (B) MR estimates for lymphocyte cell count on IgAN (East Asian). (C) MR estimates for neutrophil cell count on IgAN (East Asian). (D) MR estimates for monocyte cell count on IgAN (East Asian). (E) MR estimates for eosinophil cell count on IgAN (East Asian). (F) MR estimates for basophil cell count on IgAN (East Asian).


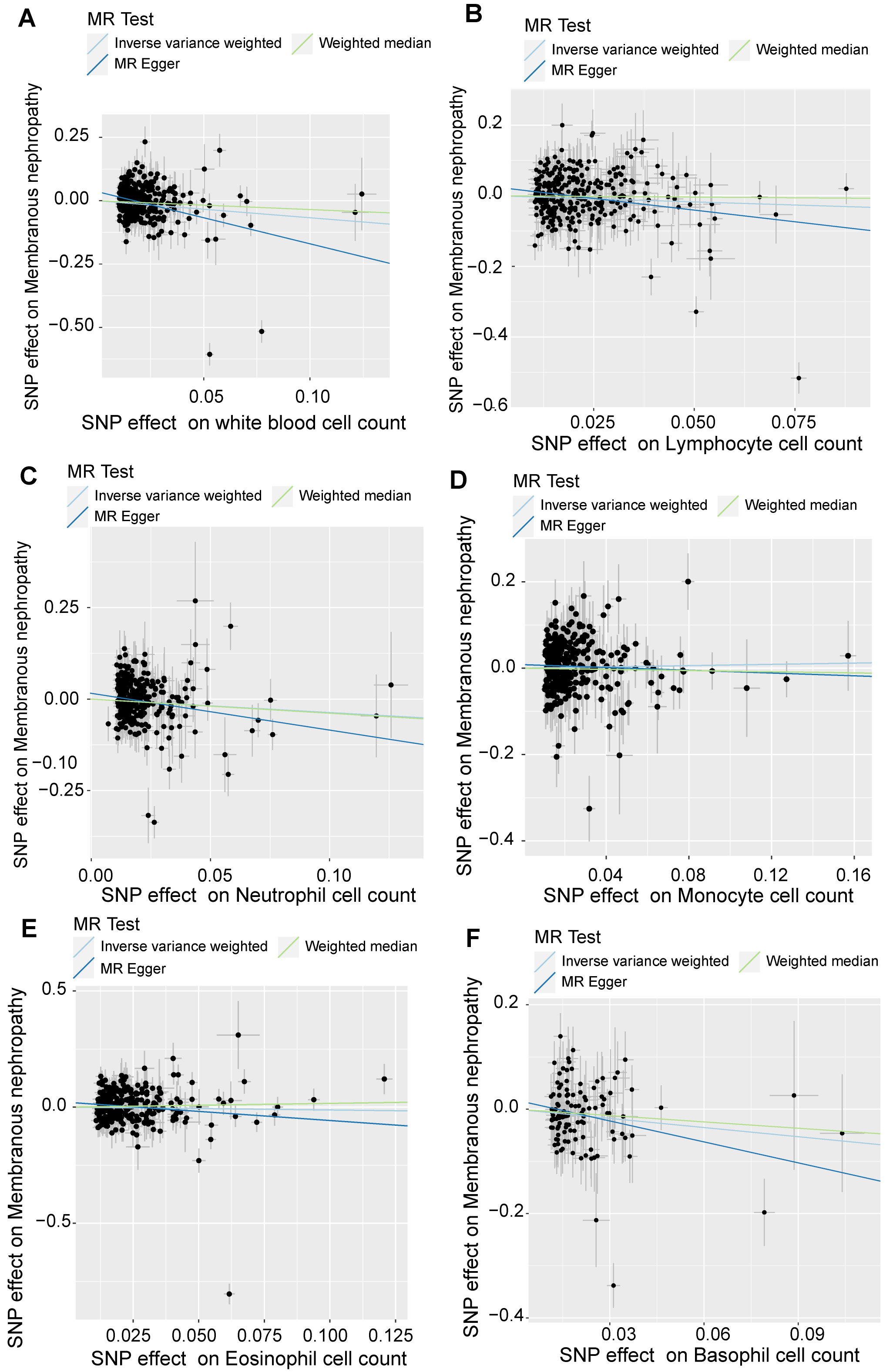


**Supplementary Figure S4.** **Scatter plot of SNPs associated with peripheral immune cells count and the risk of membranous nephropathy (MN)**. The plot presents the effect sizes of the SNP-peripheral immune cells count association (x-axis, SD units) and the SNP-membranous nephropathy association (y-axis, log (OR)) with 95% conffdence intervals. The regression slopes of the lines correspond to causal estimates using the three Mendelian randomization (MR) methods (the Inverse variance weighted method, weighted median estimator, and MR-Egger).(A)White blood cell count on membranous nephropathy; (B) Lymphocyte cell count on membranous nephropathy. (C) Neutrophil cell count on membranous nephropathy. (D) Monocyte cell count on membranous nephropathy. (E) Eosinophil cell count on membranous nephropathy. (F) Basophil cell count on membranous nephropathy.


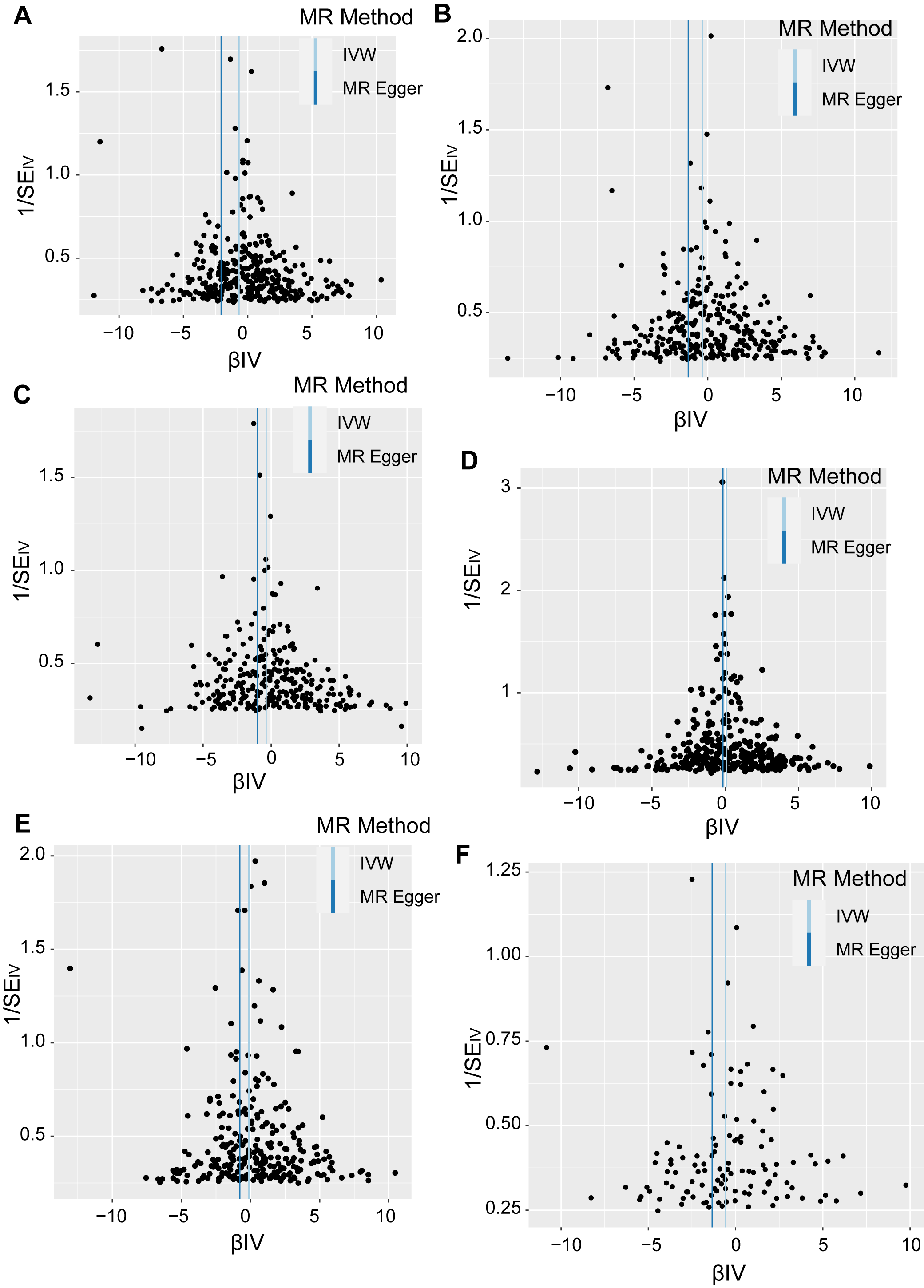


**Supplementary Figure S5.** **Funnel plot to assess heterogeneity (Membranous nephropathy, MN).** Funnel plot of SNP for MR analysis of exposures on outcomes. The light blue line represents the inverse‐variance weighted estimate. The dark blue line represents the MR-Egger estimate. (A) MR estimates for white blood cell count on membranous nephropathy. (B) MR estimates for lymphocyte cell count on membranous nephropathy. (C) MR estimates for neutrophil cell count on membranous nephropathy. (D) Monocyte cell count on membranous nephropathy. (E) MR estimates for monocyte cell count on membranous nephropathy. (F) MR estimates for basophil cell count on membranous nephropathy.


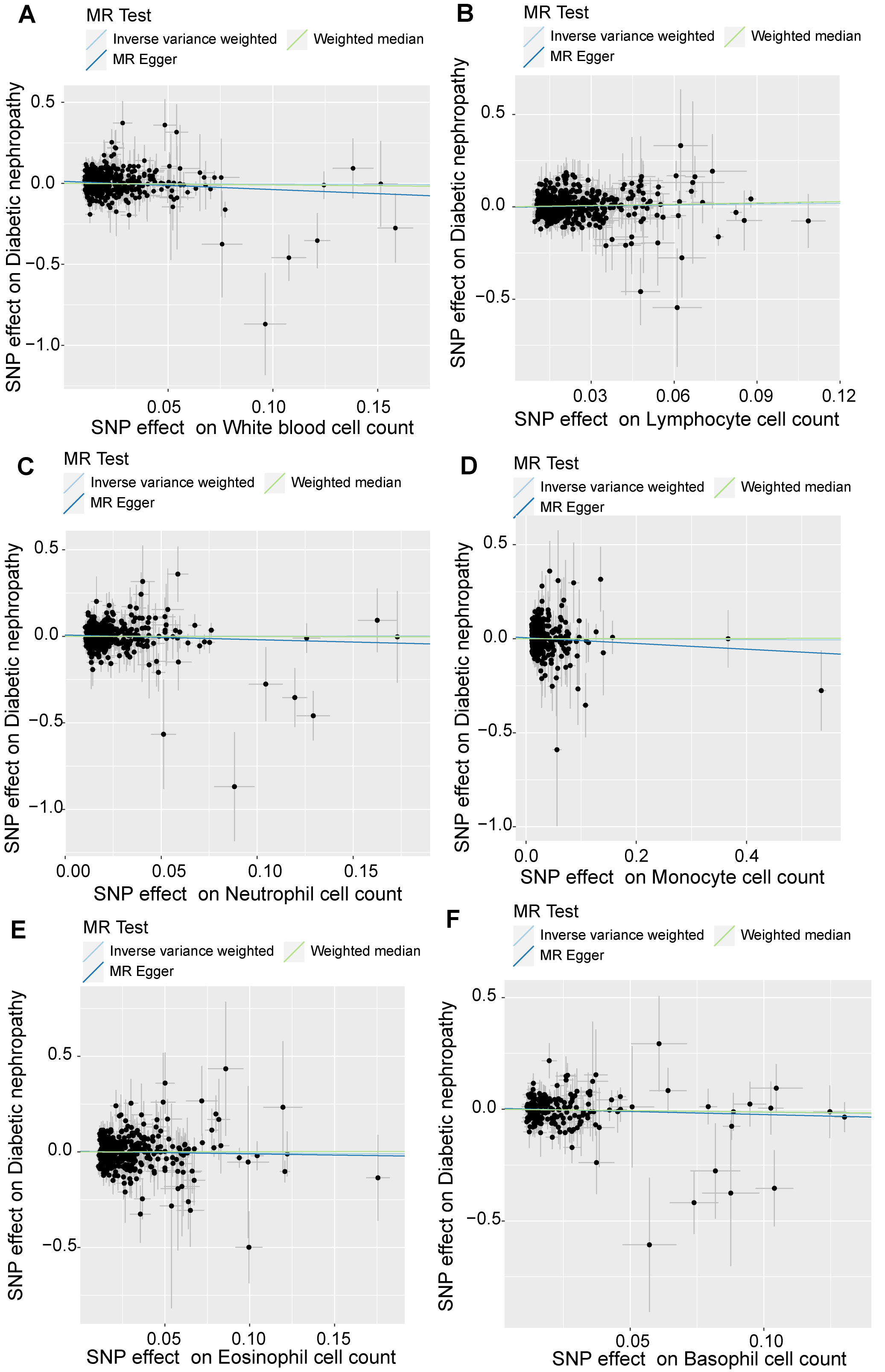


**Supplementary Figure S6. Scatter plot of SNPs associated with peripheral immune cells count and the risk of diabetic nephropathy (DN)**. The plot presents the effect sizes of the SNP-peripheral immune cells count association (x-axis, SD units) and the SNP-diabetic nephropathy association (y-axis, log (OR)) with 95% conffdence intervals. The regression slopes of the lines correspond to causal estimates using the three Mendelian randomization (MR) methods (the Inverse variance weighted method, weighted median estimator, and MR-Egger). (A)White blood cell count on diabetic nephropathy; (B) Lymphocyte cell count on diabetic nephropathy. (C) Neutrophil cell count on diabetic nephropathy. (D) Monocyte cell count on diabetic nephropathy. (E) Eosinophil cell count on diabetic nephropathy. (F) Basophil cell count on diabetic nephropathy.


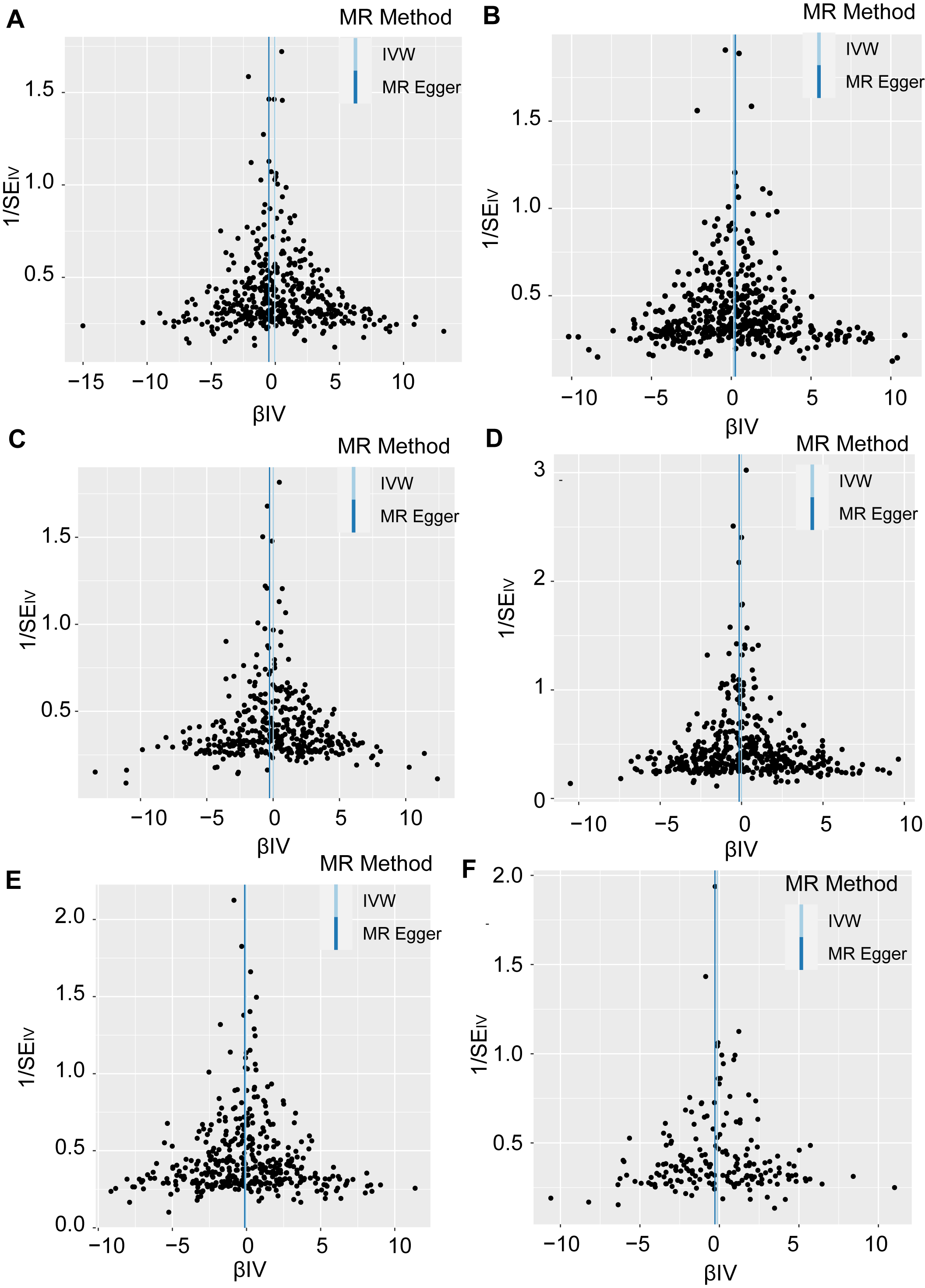


**Supplementary Figure S7.** **Funnel plot to assess heterogeneity (Diabetic nephropathy, DN).** Funnel plot of SNP for MR analysis of exposures on outcomes. The light blue line represents the inverse‐variance weighted estimate. The dark blue line represents the MR-Egger estimate. (A) MR estimates for white blood cell count on diabetic nephropathy. (B) MR estimates for lymphocyte cell count on diabetic nephropathy. (C) MR estimates for neutrophil cell count on diabetic nephropathy. (D) Monocyte cell count on diabetic nephropathy. (E) MR estimates for monocyte cell count on diabetic nephropathy. (F) MR estimates for basophil cell count on diabetic nephropathy.
